# A laboratory perspective on *Mycobacterium abscessus* biofilm culture, characterization and drug activity testing

**DOI:** 10.3389/fmicb.2024.1392606

**Published:** 2024-04-16

**Authors:** Henriëtte Margarethe Meliefste, Saskia Emily Mudde, Nicole Christine Ammerman, Jurriaan Evert M. de Steenwinkel, Hannelore Iris Bax

**Affiliations:** ^1^Department of Medical Microbiology and Infectious Diseases, Erasmus University Medical Center, Rotterdam, Netherlands; ^2^Department of Internal Medicine, Section of Infectious Diseases, Erasmus University Medical Center, Rotterdam, Netherlands

**Keywords:** *mycobacterium abscessus*, biofilm, nontuberculous mycobacteria, drug activity testing, *in vitro*, antimicrobial resistance

## Abstract

*Mycobacterium abscessus* is an emerging opportunistic pathogen causing severe pulmonary infections in patients with underlying lung disease and cystic fibrosis in particular. The rising prevalence of *M. abscessus* infections poses an alarming threat, as the success rates of available treatment options are limited. Central to this challenge is the absence of preclinical *in vitro* models that accurately mimic *in vivo* conditions and that can reliably predict treatment outcomes in patients. *M. abscessus* is notorious for its association with biofilm formation within the lung. Bacteria in biofilms are more recalcitrant to antibiotic treatment compared to planktonic bacteria, which likely contributes to the lack of correlation between preclinical drug activity testing (typically performed on planktonic bacteria) and treatment outcome. In recent years, there has been a growing interest in *M. abscessus* biofilm research. However, the absence of standardized methods for biofilm culture, biofilm characterization and drug activity testing has led to a wide spectrum of, sometimes inconsistent, findings across various studies. Factors such as strain selection, culture medium, and incubation time hugely impact biofilm development, phenotypical characteristics and antibiotic susceptibility. Additionally, a broad range of techniques are used to study *M. abscessus* biofilms, including quantification of colony-forming units, crystal violet staining and fluorescence microscopy. Yet, limitations of these techniques and the selected readouts for analysis affect study outcomes. Currently, research on the activity of conventional antibiotics, such as clarithromycin and amikacin, against *M. abscessus* biofilms yield ambiguous results, underscoring the substantial impact of experimental conditions on drug activity assessment. Beyond traditional drug activity testing, the exploration of novel anti-biofilm compounds and the improvement of *in vitro* biofilm models are ongoing. In this review, we outline the laboratory models, experimental variables and techniques that are used to study *M. abscessus* biofilms. We elaborate on the current insights of *M. abscessus* biofilm characteristics and describe the present understanding of the activity of traditional antibiotics, as well as potential novel compounds, against *M. abscessus* biofilms. Ultimately, this work contributes to the advancement of fundamental knowledge and practical applications of accurate preclinical *M. abscessus* models, thereby facilitating progress towards improved therapies for *M. abscessus* infections.

## 1 Introduction

*Mycobacterium abscessus*, belonging to the rapidly growing non-tuberculous mycobacteria, is an emerging opportunistic pathogen that can cause severe pulmonary infections in patients with underlying lung disease, such as cystic fibrosis. The prevalence of *M. abscessus* infections has been increasing over the past decades (Cristancho-Rojas et al., 2023). This is an alarming threat, as the success rates of available treatment options are limited (Chen et al., 2019). Consequently, there is an urgent need for novel treatment regimens. Central to the challenge of drug development is the lack of preclinical *in vitro* models that accurately mimic *in vivo* conditions and that can reliably predict treatment outcomes in patients.

*Mycobacterium abscessus* is increasingly recognized for its association with biofilm formation in the alveolar walls and lung cavity of patients, and on medical devices (El Helou et al., 2013; Qvist et al., 2015; Fennelly et al., 2016). It is widely acknowledged that bacteria in biofilms are more recalcitrant to antibiotic treatment compared to planktonic bacteria. For example, the biofilms’ extracellular matrix (ECM) forms a barrier that protects the bacteria from antibiotic penetration (Ortíz-Pérez et al., 2011; Singh et al., 2017). Moreover, bacteria within biofilms exhibit a heterogeneity of metabolic states, including a non-replicating state in which bacteria are able to survive low nutrient and oxygen levels, but are less susceptible to antibiotics (Singh et al., 2017). Consequently, guideline-recommended drugs might be insufficient in killing *M. abscessus* within biofilms. Hence, it is plausible that the reliability of preclinical drug activity assays will benefit from incorporating *M. abscessus* biofilm models next to the currently accepted standard assays using planktonic mycobacteria.

**Figure 1:**
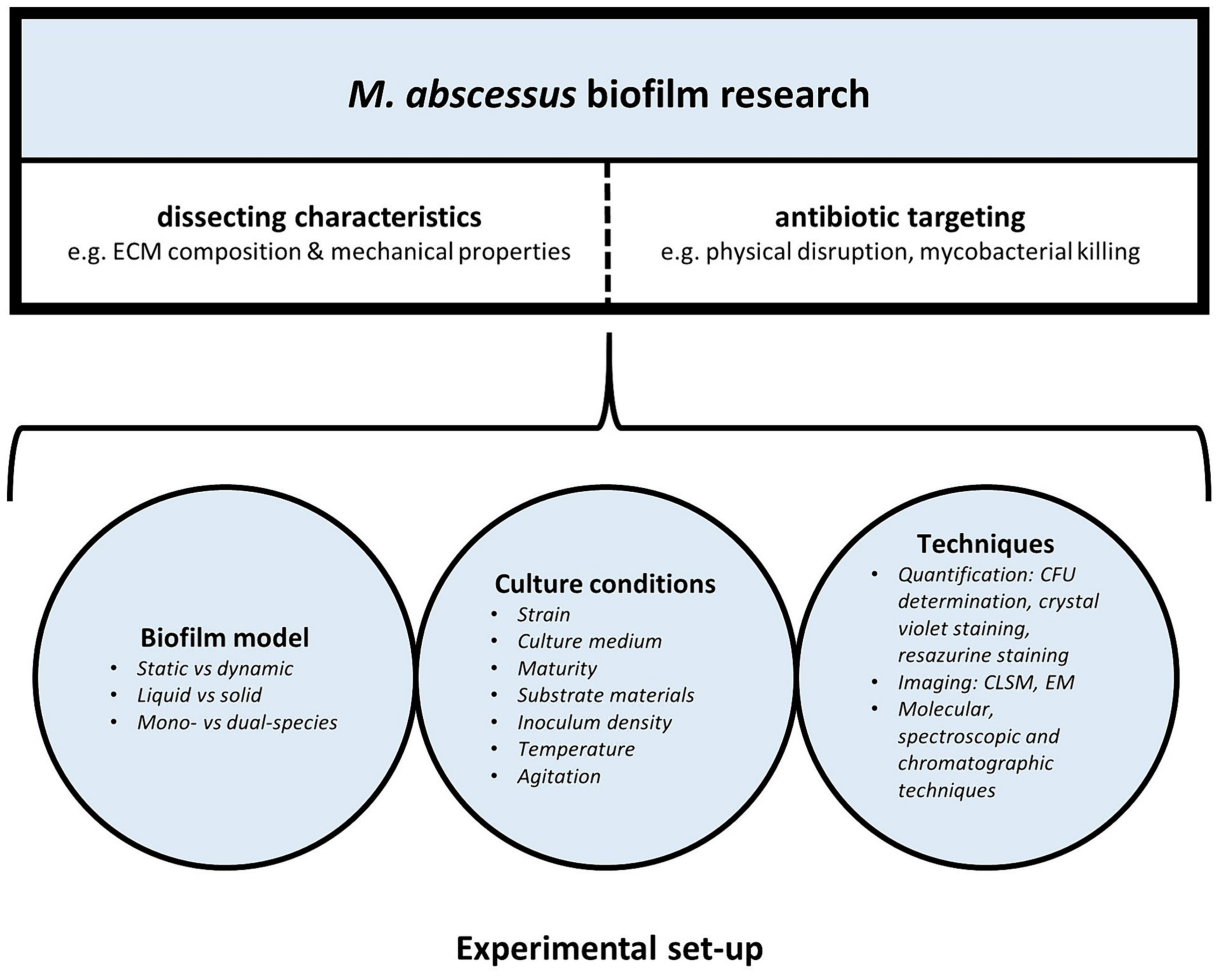
Laboratory set-up of *M. abscessus* biofilm research: models, experimental conditions, and techniques influence *M. abscessus* biofilm characteristics and behavior.

In recent years there has been a growing interest in *M. abscessus* biofilm research. However, there is no standardized method for biofilm culture, characterization and drug activity testing, hampering appropriate comparison between different studies. Both the type of biofilm model and experimental conditions used in *M. abscessus* biofilm studies can affect biofilm characteristics and behavior, contributing to the wide spectrum of seemingly inconsistent findings across various *M. abscessus* biofilm studies (Figure 1).

In this context, we provide an overview of the laboratory models and techniques that have been described to study *M. abscessus* biofilms (search strategy is provided in Supplementary material S1). We elaborate on the current insights of *M. abscessus* biofilm characteristics and describe the present understanding of the activity of traditional antibiotics, as well as potential novel compounds, against *M. abscessus* biofilms.

## 2 Building the biofilm research fundament—laboratory models used for *Mycobacterium abscessus* biofilm culture

### 2.1 Classical biofilm culture methods

Various static and dynamic culture methods have been used to culture *M. abscessus* biofilms *in vitro*, involving both liquid and solid surface biofilm culture systems. In liquid biofilm cultures, *M. abscessus* has been shown to exhibit various growth patterns, including growth at the bottom of the well, as a pellicle at the liquid-air interface, or even both submerged and as a pellicle (Clary et al., 2018; Hunt-Serracin et al., 2019; Belardinelli et al., 2021; Li et al., 2022). In fact, one study reported that *M. abscessus* formed its biofilm at the liquid-air interface when grown for 20 days in standard medium, while the biofilm submerged within 29 h when exposed to the reducing agent dithiothreitol (Chakraborty et al., 2021). This suggests that environmental factors affect *M. abscessus* biofilm behavior, although the precise factors that contribute to biofilm location remain unknown. Biofilm location is an important aspect for the reproducibility and practicability of experiments. As such, submerged biofilms are more readily separated from the planktonic population, facilitating a reliable assessment of the biofilm population. Importantly, methods to separate pellicle-biofilms from planktonic bacteria are often not described. Moreover, it could be speculated that submerged biofilms might exhibit distinct behavior patterns compared to those at the liquid-air interface due to variations in, for example, oxygen concentration and mechanical forces.

For biofilm culture in liquid medium, the most commonly used method is the static *microtiter plate method*, in which a microtiter plate is inoculated with a *M. abscessus* suspension after which a visible mycobacterial biofilm is formed over time. This relatively simple method enables simultaneous testing of multiple conditions that might affect the formation or degradation of the *M. abscessus* biofilm, facilitating high-throughput screening. However, the limited surface area of microtiter plate wells restricts the amount of biofilm that can be cultured, potentially hampering the reliability and practicability of downstream analyses. Alternatively, larger volumes have been used for biofilm growth, including polystyrene or glass test tubes or tissue culture flasks (Keefe and Bermudez, 2022). While the consequence of using larger volumes is a lower throughput, it is an advantageous method when high biomass is required for in-depth characterisation analyses, such as proteomics. Another well-established method for biofilm culture is the *MBEC (Minimal Biofilm Eradication Concentration) Assay Kit^®^* (formerly known as the Calgary Biofilm Device) (Ceri et al., 1999). This device consists of a 96-well culture plate with a plastic lid containing 96 pegs. Biofilms form on the pegs, as these pegs are partially submerged in inoculated culture medium. After biofilm formation these pegs can easily be transferred to another 96-wells plate containing a range of antibiotic concentrations, allowing the determination of the minimal biofilm eradication concentration by visual observation or by colony-forming units (CFU) determination, making it a more standardized approach compared to the microtiter plate method. Additionally, *M. abscessus* biofilms can be cultured in *chambered slides* (Muñoz-Egea et al., 2016; Chakraborty et al., 2021; Yue et al., 2022; Kurbatfinski et al., 2023). With this method the biofilm is grown in individual wells on a chamber slide system, allowing for direct visualization of live bacteria using microscopy. As such, chamber slides are suitable for monitoring biofilm maturation and assessing the activity of antimicrobial agents on the biofilm in real-time. Another method described for *M. abscessus* biofilm culture involves the addition of *glass beads* to the culture medium (Falkinham et al., 2012; Oschmann-Kadenbach et al., 2024). The biofilm forms on the glass beads after which the bacteria can be obtained by vortexing.

Besides the above-mentioned static biofilm models, more dynamic models have been developed for biofilm research, in which biofilms can be cultured with a continuous flow of culture medium. Biofilms cultured in a *chamber flow cell* can be easily stained and imaged, allowing for real-time monitoring of the biofilm under dynamic conditions (Malcolm et al., 2013). In addition, the *Centre for Disease Control biofilm reactor*, comprises a vessel equipped with multiple coupons (Goeres et al., 2005). The vessel can be filled with culture medium submerging each coupon, and two pumps facilitate the in- and outflow of medium. The coupons are easily obtained for CFU determination and are available in various materials, allowing the evaluation of various substrate materials on biofilm formation (Mullis and Falkinham, 2013).

An alternative method for growing biofilms in liquid medium is by using *a colony-forming model*, in which the biofilm is grown on a solid surface—typically by placing an inoculated semi-permeable membrane on top of solid agar (Rodríguez-Sevilla et al., 2018a; Gloag et al., 2021; Aguilera-Correa et al., 2023). Biofilms can be detached and collected via sonicating and vortexing the membrane. An important advantage of this model is that biofilm transfer to fresh medium or to medium containing antimicrobials is relatively straightforward. There are no washing steps needed, which is beneficial for fragile biofilms like those formed by *M. abscessus*. Moreover, both sides of the biofilm can be accessed, allowing various analyses, such as the antibiotic permeability assay (Ortíz-Pérez et al., 2011). Colony-forming biofilms share morphological traits of liquid grown biofilms and tend to have more biomass, enabling the study of various aspects, such as its biomechanical properties (Gloag et al., 2021). In addition, the colony-forming biofilm model has been used to study the interaction of *M. abscessus* and *Pseudomonas aeruginosa* in a polymicrobial biofilm, and to analyse the growth of smooth and rough *M. abscessus* biofilms in response to antibiotic exposure (Rodríguez-Sevilla et al., 2018a, 2019; Idosa et al., 2022; Aguilera-Correa et al., 2023).

### 2.2 Mimicking the *Mycobacterium abscessus* lung niche: more complex biofilm models

During biofilm formation in humans, it has been postulated that *M. abscessus* interacts with host immune factors and other bacterial communities (Park et al., 2021; Idosa et al., 2022). Interestingly, interaction between bacterial species can result in structural and functional changes within the biofilm, leading to increased bacterial resistance to antibiotics (Burmølle et al., 2014). *M. abscessus* can be co-isolated with *P. aeruginosa*, another major pathogen in cystic fibrosis patients. Therefore, a *dual-species biofilm model* was developed to explore the interaction between *M. abscessus* and *P. aeruginosa* (Adjemian et al., 2018). In this model, a stable colony-forming dual-biofilm is formed within 48–72 h on tryptic soy agar, which is suitable for analyses such as microscopy and CFU determination (Rodríguez-Sevilla et al., 2018a). Later, a similar model was developed using 7H10 Middlebrook agar as culture medium (Idosa et al., 2022). In comparison to *M. abscessus, P. aeruginosa* has a high growth rate, resulting in a greater abundance of *P. aeruginosa* relative to *M. abscessus* within dual-species biofilms (Rodríguez-Sevilla et al., 2018a). Two studies observed that co-culturing *M. abscessus* with *P. aeruginosa* caused a reduction in the number of *M. abscessus* compared to a single-species *M. abscessus* biofilm (Rodríguez-Sevilla et al., 2018a; Idosa et al., 2022). Thus, *P. aeruginosa* could have a competitive advantage over *M. abscessus*, thereby affecting the biofilm structure and characteristics, which could also be the case for other pathogens that are co-isolated in patients with *M. abscessus* infections. Interestingly, *M. abscessus* and *P. aeruginosa* co-aggregate, yet their spatial distribution within the biofilm seems to differ. *M. abscessus* was found to predominantly localize in the inner and lower zone of the biofilm, whereas *P. aeruginosa* also grew in the upper layers (Rodríguez-Sevilla et al., 2018a). The direct dynamics between *M. abscessus* and *P. aeruginosa* within biofilms are still rather unexplored and probably involve a complex interplay. Overall, the dual-species biofilm model provides important additional information compared to the traditional single-species biofilm, as selectively targeting one species can give a competitive advantage to the other, hereby altering the overall biofilm dynamics (Rodríguez-Sevilla et al., 2019).

To more closely mimic host-pathogen interactions, a *3D lung epithelial model* was developed in which *M. abscessus* was able to form a single- and dual-species biofilm with *P. aeruginosa* on lung epithelial cells (Rodríguez-Sevilla et al., 2018b). Moreover, *M. abscessus* biofilm formation was shown in *human airway organoids* (Leon-Icaza et al., 2023). In another model, *neutrophils* were adhered to coverslips in flow cells, after which *M. abscessus* biofilm was formed (Malcolm et al., 2013). Interestingly, biofilm density was enhanced by neutrophils (Malcolm et al., 2013). Ultimately, such models could be used to study the interactions between host cells and bacterial responses to antibiotics.

## 3 Culture condition complexities—an overview of experimental variables

The absence of standardized biofilm culture methods introduces technical variabilities across studies, including differences in strain selection, culture medium, and biofilm maturity.

### 3.1 Strain selection

Different *M. abscessus* strains are used in biofilm research. This is important as differences in biofilm formation capability and robustness have been described among different isolates (Martín-de-Hijas et al., 2009; Saptawati et al., 2022). Yet, the factors contributing to this (sub) strain variability and the extent to which this variability affects biofilm formation and behavior remain underexplored. Studies on *M. abscessus* biofilms are most commonly performed using the ATCC 19977 strain, an *M. abscessus* subsp. *abscessus*, due to its widespread availability and its relatively well-established work protocols. Advantageously, the genome of the ATCC 19977 strain is similar to the genomes of other relevant (clinical) isolates (Davidson et al., 2014; Davidson, 2018). Other *M. abscessus* subspecies have only occasionally been used in biofilm research (Table 1). It could be speculated that genetic differences among *M. abscessus* subspecies may affect biofilm formation and characteristics. There are studies on *M. abscessus* biofilms and drug activity testing that include multiple isolates, although their primary focus was not on strain variability. These studies showed no major differences between the isolates in terms of drug susceptibility (Ortíz-Pérez et al., 2011; Rodríguez-Sevilla et al., 2018a; Marini et al., 2019; Rodríguez-Sevilla et al., 2019; Belardinelli et al., 2022a; Feizi et al., 2023). Nevertheless, it may be worthwhile to further unravel strain-specific biofilm behavior, including antibiotic susceptibility, given the paucity of data on this topic.

**Table 1:** Overview of *M. abscessus* isolates and the corresponding number of studies including each isolate for *M. abscessus* biofilm research.

Strain	Number of studies
*M. abscessus* subsp. *abscessus* ATCC 19977	37
*M. abscessus* subsp. *abscessus* Bamboo	3
*M. abscessus* subsp. *bolletii* 101	1
*M. abscessus* subsp. *bolletii* CIP 108541	1
*M. abscessus* subsp. *bolletii* NR-44261	1
*M. abscessus* subsp. *massiliense* GO06	1
*M. abscessus* subsp. *massiliense* CIP 108297	1
Clinical isolate (no subspecies mentioned)	19
Clinical isolate (subsp. *massiliense*)	3
Clinical isolate (subsp. *abscessus*)	2
Not described	3

Another important consideration regarding strain selection is the distinction between the smooth and rough morphotype that *M. abscessus* exhibits. Initially it was thought that only smooth *M. abscessus* morphotypes were capable of forming biofilms (Howard et al., 2006; Greendyke and Byrd, 2008; Bernut et al., 2016). However, subsequent studies demonstrated that the rough morphotype, which differs from the smooth morphotype by absence of glycopeptidolipids in the cell wall, was also able to form biofilms (Howard et al., 2006; Williams et al., 2009; Clary et al., 2018; Gloag et al., 2021; Aguilera-Correa et al., 2023; Veigyabati Devi and Singh, 2023; Oschmann-Kadenbach et al., 2024). This discrepancy is likely due to differences in experimental conditions, experimental duration and strains, further emphasizing the importance of using different experimental variables in *M. abscessus* biofilm research (Clary et al., 2018). The smooth and rough morphotype biofilms have distinct characteristics, including differences in macroscopic and microscopic appearances (Williams et al., 2009; Clary et al., 2018; Gloag et al., 2020; Born et al., 2023). For example, the smooth morphotype colony biofilm has a smooth viscous appearance whilst the rough morphotype has a fractal-like appearance on a macroscopic level (Gloag et al., 2021). In liquid culture, the smooth and rough morphotype biofilms were, respectively, described as “oleaginous” and “waxy” (Clary et al., 2018). In addition, smooth and rough morphotype biofilms also differ in their viscoelastic properties (section 5.1.5) (Gloag et al., 2021). Whether the smooth and rough morphotype biofilms also differ in other aspects, such as ECM composition or its interaction with host cells is unknown.

### 3.2 Culture medium

*M. abscessus* can form biofilms in different types of culture medium. The most commonly used media are 7H9 Middlebrook medium, Sauton’s medium and Synthetic Cystic Fibrosis Medium (SCFM) (Table 2). These media differ in chemical composition, pH, viscosity, and nutrient availability, which could impact biofilm formation. In fact, media dependent differences in macroscopic appearance as well as biochemical composition of *M. abscessus* biofilms have been described (Hunt-Serracin et al., 2019; Keefe and Bermudez, 2022). Of note, some studies specified the omission of Tween 80, a common supplement in 7H9 Middlebrook broth, from their culture medium. Tween 80 inhibits aggregation of *M. abscessus*, and omission of Tween 80 might therefore promote biofilm formation (Kolpen et al., 2020). However, it could be argued that the presence of a detergent such as Tween 80 in culture medium might actually mimic the surface tension reducing function of lung surfactant in the *in vivo* situation. In this context, the exact influence of lung surfactant on *M. abscessus* biofilm formation is unclear. It could be speculated that lung surfactant might inhibit biofilm formation, indicated by the observation that the phospholipid dioleoylphosphatidylcholine (DOPC) slowed down *M. abscessus’* surface attachment *in vitro* (Belardinelli et al., 2021). Whether this also holds true for the *in vivo* situation is currently unknown, especially since not DOPC, but dipalmitoylphosphatidylcholine (DPPC), is the most abundant phosphatidylchole present in lung surfactant (King, 1982). Alternatively, it has been shown for bacteria other than *M. abscessus* that components of lung surfactant were able to induce a stress response in bacteria which facilitated biofilm formation (Willsey et al., 2018). It is however unclear how those findings relate to *M. abscessus*. In addition to culture medium, specific components have been reported to affect biofilm growth. In this context, it was previously suggested that iron plays a role in *M. abscessus* biofilm formation, as a ferritin gene knock out in *M. abscessus* resulted in decreased biofilm formation (Pereira et al., 2023). In addition, omitting FeSO4 from SCFM led to decreased biofilm biomass compared to complete SCFM (Belardinelli et al., 2021). Other specific cations could affect biofilm formation as well. For example, the omission of magnesium from SCFM medium significantly decreased biofilm mass, and RNA sequencing revealed uniquely expressed enzymes in both SCFM- and Hank’s Balanced Salt Solutions-grown biofilms that depended on magnesium as a co-factor (Belardinelli et al., 2021; Keefe and Bermudez, 2022). However, since supplementing SCFM with magnesium did not result in increased biofilm mass, its exact role in biofilm formation has yet to be elucidated (Belardinelli et al., 2021). The importance of calcium in biofilm formation is unclear. Although its presence was critical for *M. abscessus* biofilm formation in a colony forming model, other studies using a liquid biofilm model could not confirm these findings (Belardinelli et al., 2021; Keefe and Bermudez, 2022). These inconsistencies might be attributable to the presence of alternative nutrients in the various media that might have acted as calcium substitutes. Of note, to determine the exact effect of medium components on biofilm formation, rather than on mycobacterial growth alone, inclusion of appropriate planktonic controls is required.

**Table 2:** Overview of culture media and the corresponding number of studies including each medium for *M. abscessus* biofilm research.

	Number of studies
*Liquid culture medium*	
7H9 Middlebrook broth	11
7H9 Middlebrook broth without Tween 80	5
7H9 Middlebrook broth + OADC	4
7H9 Middlebrook broth +10% OADC +0.5% glycerol	2
Sauton’s medium	10
Sauton’s medium +10% ADC	1
Synthetic Cystic Fibrosis Medium	6
Mueller-Hinton broth	1
GTSF-2 medium +2.5 mg/L ITS +1.5 g/L sodium bicarbonate +10% FBS*	1
Hartmans-de Bont broth	1
Iscove’s medium*	1
Lysogeny broth + glucose	1
M63 salts + glucose + CaCl_2_ + MgSO_2_	1
RPMI +10% heat inactivated FBS*	2
Tryptic soy broth	1
Hanks’ balanced salt solution	1
Unclear	3
*Solid culture medium*	
Tryptic soy agar +5% sheep blood	3
7H10 Middlebrook agar + OADC	2
7H11 Middlebrook agar	1
B4 biofilm-promoting solid medium + calcium acetate or EDTA.	1
Mueller-Hinton agar containing antibiotics	1
Solid Synthetic Cystic Fibrosis Medium	1

Cell culture media are indicated with *. OADC, Oleic Albumin Dextrose Catalase; ADC, albumin, dextrose, catalase ITS, Insulin-Transferrin-Selenium; FBS, fetal bovine serum; RPMI, Roswell Park Memorial Institute; EDTA, Ethylenediamine tetraacetic acid.

Besides considering the medium type and composition, maintaining a static environment versus continuous replenishing of medium is a factor that varies across studies and that could impact biofilm formation. Daily replenishment can provide essential nutrients to support biofilm formation, while maintaining the medium could lead to nutrient depletion, potentially mimicking *in vivo* site-of-infection environments and altering biofilm behavior.

### 3.3 Biofilm maturity

The duration of biofilm culture before starting experiments varies widely among studies, ranging from 6 h to 28 days, which might affect biofilm characteristics (Supplementary Table S1) (Belardinelli et al., 2021; Dokic et al., 2021). Biofilm formation is generally distinguished into 3 phases, including an early, middle and mature stage. Although there is no consensus on the exact duration of each phase for *M. abscessus* biofilm formation, the early phase is typically considered at day 1 to 2, the middle phase at day 3 to 5 and the mature phase at day 6 to 7 (Belardinelli et al., 2021; Dokic et al., 2021; Veigyabati Devi and Singh, 2023). Mature biofilms are often more complex in structure and composition, although prolonged culture duration has been reported to result in decreased biofilm density, decreased bacterial viability, and biofilm dispersal (Belardinelli et al., 2021).

Biofilm maturation has been linked to a shift in gene expression (Belardinelli et al., 2021; Dokic et al., 2021). During the early-stage of surface attachment, an upregulation of genes related to membrane transport and transcription processes was observed compared to planktonic bacteria (Belardinelli et al., 2021). In the middle-stage of biofilm formation, *M. abscessus* upregulated pathways related to translation and transcription processes, along with the upregulation of several chaperones (Belardinelli et al., 2021). In addition, pathways related to fatty acid, glyoxylate and redox metabolism as well as the stress response were upregulated (Dokic et al., 2021). In the final mature stage, substantially more differentially expressed genes have been identified compared to the middle-stage biofilm. Pathways related to cell division, cell wall synthesis, transport, and energy production were reported to be downregulated, with upregulation observed for pathways related to oxidoreductase activity and propanoate metabolism indicating a genetic and metabolic shift towards a dispersal-ready state of the biofilm (Dokic et al., 2021). Overall, a dynamic shift in the expression of genes and metabolic pathways has been described, reflecting the evolving cellular activities during biofilm development. The process of changing gene expression during the different stages of biofilm formation was found to be carefully orchestrated by several transcription regulators, including members from the Tetracycline Resistance Element Repressor and Multiple Antibiotic Resistance Regulator family (both associated with biofilm formation and virulence in other bacteria) (Veigyabati Devi and Singh, 2023). Studying the unique expression of transcripts and transcriptional regulators at specific stages of the biofilm cycle is important as potential therapeutic targets might be identified.

### 3.4 Others

Several additional experimental variables that might affect *M. abscessus* biofilm formation are important to consider when designing *M. abscessus* biofilm experiments. First, variability in substrate materials has been reported. These substrates might differ in surface texture, hydrophobicity, chemical composition and porosity. While the exact influence of these (subtle) material differences are unclear, it is recognized that biofilm adherence may vary depending on the material. For example, better adherence of *M. abscessus* to glass compared to polyvinyl chloride has been reported (Mullis and Falkinham, 2013). Second, different culture volumes have been reported across studies, which might affect experimental conditions such as nutrient and oxygen availability. Third, the density of the mycobacterial starting inoculum, ranging from 10^5^ CFU/mL to 10^9^ CFU/mL in *M. abscessus* biofilm studies, might affect biofilm formation since higher inoculum densities might expedite biofilm formation, increase the competition for nutrients, affect inter-microbial interactions, and potentially impact the final density and thickness of a mature biofilm (Stepanović et al., 2003; Rodríguez-Sevilla et al., 2018a; Lichtenberg et al., 2022). In addition, the method of inoculum preparation—either derived from liquid or solid medium—might play a role in biofilm formation, as was observed for bacteria other than *M. abscessus* (Kiers et al., 2001; Stepanović et al., 2007). This might be due to the differential expression of cell-associated molecules related to adhesion, an important process during the initial stages of biofilm development, in solid grown bacteria compared to liquid grown bacteria (Kiers et al., 2001; Stepanović et al., 2007). Fourth, experimental temperature might impact biofilm formation. While the most commonly reported temperature used in *M. abscessus* biofilm experiments was 37°C, reflecting the human body temperature, studies vary in temperature settings from room temperature to 37°C. Yet a fifth factor is culture agitation. Most studies induced biofilm formation without culture agitation. Remarkably, some studies induced biofilm formation under agitating conditions, with a speed ranging from 70 to 150 rpm. This could potentially impact nutrient and oxygen diffusion, as well as affect the mechanical load on the biofilm (Greendyke and Byrd, 2008; Muñoz-Egea et al., 2016; Feizi et al., 2023).

## 4 Exploring the toolbox—commonly used techniques for studying and analysing *Mycobacterium abscessus* biofilms

A variety of techniques are used for studying *M. abscessus* biofilms, offering insights into the biofilm growth dynamics, structure, mechanical properties, and response to antibiotics.

### 4.1 Biofilm quantification

*CFU determination* is the most commonly used method for quantifying the number of viable bacteria within *M. abscessus* biofilms (Supplementary Table S1). In this case, the biofilm is homogenized, after which the biofilm suspension is serially diluted and plated onto agar for subsequent colony counting after several days of incubation. CFU determination is used to assess absolute and relative changes in CFU values over time, and to assess the activity of mycobacterial drugs. Although CFU determination within biofilms is a relatively accessible method, since it does not require specialized equipment, it comes with challenges, as there is a high risk of bacterial clumping due to the presence of an ECM that facilitates bacterial aggregation (Hunt-Serracin et al., 2019). This can lead to an underestimation of the true viable population. Another challenge of CFU determination is that biofilms have different microenvironments, including regions with low nutrient and oxygen availability, in which bacteria may enter a viable but non-culturable state (Li et al., 2014). This can also lead to an underestimation of the viable population. Thus, although CFU determination is a valuable tool, it is essential to be mindful of these considerations influencing accuracy and reliability of the results obtained. As such, combining CFU results with other readouts should provide a more comprehensive understanding of the *M. abscessus* biofilm.

*Crystal violet staining* is a commonly used method to quantify biofilm biomass. Crystal violet is a cationic dye that penetrates the biofilm structure and binds to negatively charged components of the biofilm. Although it is debatable to what extent crystal violet stains the actual mycobacteria, crystal violet does stain crucial ECM components of the *M. abscessus* biofilm, including polysaccharides, extracellular DNA (eDNA), and proteins. The staining can be quantified by solubilizing the crystal violet with an extraction buffer and measuring the intensity of the absorbed light using a microplate reader. The intensity of the staining correlates with biofilm mass and can be expressed as absolute optical density values or relative biomass. Beyond its role in biofilm quantification, crystal violet staining can also facilitate the macroscopic visualization of the biofilm. Similar to crystal violet, *safranin staining* binds negatively charged molecules and is used for the same purpose as crystal violet, although it is less commonly used. A disadvantage of both staining techniques includes their non-specificity. For example, they do not distinguish between live and dead bacteria.

Another method described for biofilm quantification is the use of *resazurine* or *Tetrazolium chloride (XTT)* (Pettit et al., 2005; Marini et al., 2019). Resazurine is a blue-colored dye that is absorbed by living cells, where it is enzymatically reduced into the fluorescent pink-colored molecule resorufin. Since the fluorescence of resorufin is proportional to the metabolic activity and viability of cells, this stain evaluates the viability of cells within the biofilm. Fluorescence signals obtained by measuring biofilm samples in a microtiter plate reader can be used to estimate the inhibitory activity of compounds against *M. abscessus* residing in biofilms (Belardinelli et al., 2021; Lee et al., 2021, 2022; Feizi et al., 2023). In addition, macroscopic images of the biofilm wells can be obtained allowing rapid colorimetric assessment. Resazurine has the limitation that it specifically identifies metabolically active cells but may not detect non-metabolically active bacteria with intact membranes. Consequently, resazurin serves as a measure of the metabolic activity of the bacterial population rather than as a direct outcome measure for bacterial load. This is particularly important to consider when using resazurine staining in drug activity experiments, as drugs that target protein synthesis can reduce enzyme activity, resulting in less resazurine conversion, without necessarily killing bacteria. Morever, since bacteria in biofilms exhibit a heterogeneity of metabolic states, resazurine staining can result in mixed signals (Sandberg et al., 2009). Another pitfall of this allegedly quantification method is that low bacterial concentrations within the biofilm may results in signals below the lower limit of detection (Sandberg et al., 2009). XTT operates in a manner similar to resazurine, in which yellow salt is reduced by metabolically active bacteria into the orange formazan. Besides XTT, there are other tetrazolium salts available, although these have not yet been utilized for studying *M. abscessus* biofilms.

Finally, in some studies *M. abscessus* is transfected with a *fluorescent protein*, such as mCherry (Clary et al., 2018; Belardinelli et al., 2021, 2022b). This allows the measurement of the relative fluorescence intensity of mycobacteria within biofilms, not only providing insight into the density of bacterial populations, but also into the spatial distribution of the bacteria. Recently, mWasabi transfected *M. abscessus* in a colony-biofilm was photographed using a fluorescence microscope, and the intensity of fluorescence in different areas of the images was measured over time (Aguilera-Correa et al., 2023). Subsequently, the volume of each colony was calculated with a mathematical formula based on the signal intensity and colony size. This approach mirrored the CFU counts, and thus provided a rapid estimation of the number of bacteria in the biofilm (Aguilera-Correa et al., 2023).

### 4.2 Biofilm imaging

*Mycobacterium abscessus* biofilm imaging provides insight into biofilm structure, organization, and composition. First, simple bright-field light microscopy can be used in combination with Ziehl-Neelsen acid fast staining, which has been used to show the aggregative nature of *M. abscessus* and to differentiate *M. abscessus* from *P. aeruginosa* in a dual-species biofilm (Rodríguez-Sevilla et al., 2018a; Kolpen et al., 2020). Additional techniques are required for visualizing the ECM of the biofilm, such as *fluorescence microscopy* or *scanning electron microscopy*. Fluorescence microscopy is either performed with a wide field microscope, that yields two-dimensional images, or a confocal laser scanning microscope (CLSM), that yields three-dimensional images with a high resolution allowing the visualization of different layers and regions within the biofilm. This way, CLSM could also be used for biofilm quantification, enabling the assessment of biofilm thickness and biofilm biomass. Various stainings are used for visualizing the ECM, each targeting specific ECM components, including Nile Red (lipids), TOTO-1 iodide (nucleic acids), SYBR Green 1 (nucleic acids), FilmTracer SYPRO (proteins), SYPRO Ruby (proteins), Texas Red Hydrazide (polysaccharides), and Concanavalin A (polysaccharides) (Belardinelli et al., 2021; Chakraborty et al., 2021; Dokic et al., 2021). Furthermore, Syto-9 and propidium iodide are commonly used to stain live and dead bacteria, respectively. Since Syto-9 and propidium iodide stain nucleic acids, not only the DNA of bacteria within the biofilm will be stained, but also eDNA. Propidium iodide has also been used to study the effect of genetic alternations or compounds on the amount of eDNA by adding propidium iodide directly to a biofilm culture followed by quantification of the fluorescence signal using a microplate reader (Rose and Bermudez, 2016). Since the current staining techniques allow for the detection of specific targeted ECM components only, capturing a complete image of the entire ECM might not yet be achievable. Electron microscopy is used to analyse the structure of the biofilm, the abundance of outer matrix, bacterial organization, mycobacterial density and the activity of various mycobacterial drugs or conditions with a higher spatial resolution than CLSM. In this manner, the microstructure of biofilms can be identified (Dokic et al., 2021; Li et al., 2022; Saptawati et al., 2022; Yue et al., 2022; He et al., 2023). Finally, *micro–CT X-ray* has been used to visualize the three-dimensional structures of *M. abscessus* biofilms (Cohen-Cymberknoh et al., 2022).

### 4.3 Other biofilm profiling techniques

Several metabolic, spectroscopic and chromatographic techniques have been reported for studying *M. abscessus* biofilm characteristics. First, *M. abscessus* gene expression has been evaluated by *RNA sequencing*, which gives a genome-wide overview of gene expression. Alternatively, *qRT-PCR* has been used as a more targeted approach to study the expression of specific transcripts, which can also be applied to validate results found with RNA sequencing. RNA sequencing has been used to study differentially expressed genes in young and mature *M. abscessus* biofilms compared to a planktonic population (as described in section 3.3) (Belardinelli et al., 2021; Dokic et al., 2021). Moreover, RNA sequencing has been used to study the effect of an antimicrobial peptide on transcripts to obtain a more comprehensive understanding on the peptide’s mechanism of action (Li et al., 2022). Additionally, qPCR has been used to specifically study the expression of various transcriptional regions during different specific maturity stages (Veigyabati Devi and Singh, 2023). Second, *Fourier transform infrared spectroscopy* is a non-destructive analytical technique that allows for studying and identifying the chemical composition and structures of molecules. In this context, Fourier transform infrared spectroscopy demonstrated the presence of calcium carbonate minerals and cellulose in the ECM of *M. abscessus* biofilms (Chakraborty et al., 2021; Cohen-Cymberknoh et al., 2022). Third, *mass spectrometry* can be applied to identify and quantify proteins. This technique has been used to compare protein pathways between planktonic *M. abscessus* and *M. abscessus* residing in biofilms (Rojony et al., 2020). Furthermore, proteomics has been used to assess the effect of different media on protein abundance in biofilms (section 3.2) and to examine alterations in protein compositions when biofilms are exposed to antibiotics (Rojony et al., 2020; Keefe and Bermudez, 2022). Lastly, lipids within the ECM can be analysed via *thin-layer chromatography* (see section 5.2.1) (Hunt-Serracin et al., 2019; Belardinelli et al., 2021; Dokic et al., 2021).

## 5 Integrating biofilm techniques: dissecting the extracellular matrix’ composition and mechanical properties

### 5.1 Composition of the extracellular matrix

Mycobacteria within the biofilm are embedded in an ECM, which consists of multiple components that have a structural as well as a functional role (Karygianni et al., 2020). Studying the ECM is important to gain insights into potential novel therapeutic targets and is usually performed using a combination of several of the techniques described above.

#### 5.1.1 Nucleic acids

Nucleic acids have repeatedly been reported as a fundamental part of biofilms (Karygianni et al., 2020). Multiple functions have been attributed to eDNA in biofilms, including adhesion, structural integrity, aggregation, and nutrient provision (Ibáñez de Aldecoa et al., 2017; Karygianni et al., 2020). Indeed, eDNA is reported as an important constituent of *M. abscessus* biofilms across various culture media and subspecies (Rose et al., 2015; Belardinelli et al., 2021; Chakraborty et al., 2021; Dokic et al., 2021; Oschmann-Kadenbach et al., 2024). An increase in eDNA, likely actively released, has been linked to biofilm maturation (Belardinelli et al., 2021; Ilinov et al., 2021). The increase in eDNA during biofilm maturation was found to be more pronounced compared to other constituents of the ECM, such as sugars and proteins (Belardinelli et al., 2021). eDNA was reported to be dispersed throughout the *M. abscessus* biofilm, mainly in regions with a low bacterial density, suggesting a structural role for eDNA in the *M. abscessus* biofilm by facilitating cellular aggregation. In line, exposure of biofilms to DNase caused dispersion of SCFM-cultured biofilm, although this was not confirmed when biofilms induced by thiol reductive stress were exposed to DNase (Belardinelli et al., 2021; Chakraborty et al., 2021). These contrasting findings may be due to differences in culture medium, exposure duration and biofilm maturation, indicating the role of eDNA in biofilm structure might be context-dependent (Chakraborty et al., 2021). The origin and release process of the DNA in the ECM of *M. abscessus* biofilms is yet an unexplored topic. Understanding this process might provide insights into factors that trigger or control ECM formation. For example, in *M. avium* biofilms genomic DNA is thought to be exported via nonlytic mechanisms. Sodium carbonate has been identified as a trigger for this eDNA release, which is also observed for *M. abscessus* (Rose and Bermudez, 2016). Because of the notable role of eDNA in the *M. abscessus* biofilm, eDNA might be a potential novel drug target (Rose et al., 2015).

#### 5.1.2 Lipids

Lipids in biofilm ECM are believed to contribute to adhesion, cohesion, protection and immune evasion (Karygianni et al., 2020). In *M. abscessus* biofilm, lipids are present on the surface of bacteria as well as in the inter-bacterial space (Belardinelli et al., 2021; Dokic et al., 2021). *M. abscessus* was found to be able to incorporate lipids from the environment into the ECM, in addition to actively producing and secreting endogenous lipids (Belardinelli et al., 2021; Dokic et al., 2021). For example, the phospholipid 1,2-Dioleoyl-sn-glycero-3-phosphocholine, a constituent of SCFM, was most abundantly present in the ECM of SCFM grown biofilms, next to endogenous lipids (e.g., cardiolipin) (Belardinelli et al., 2021). The majority of biofilm ECM lipids are comparable to the lipids found on the surface of planktonic cultures, although there are some lipids that are specific to the ECM of biofilms, including some types of glycerophospholipids, phosphatidylinositol dimannosides, and free mycolic acids (Belardinelli et al., 2021; Dokic et al., 2021). In fact, modifications in the chemical structure of mycolic acids during the transformation from planktonic cultures to *M. abscessus* biofilm structures have been observed in Sauton’s medium (Dokic et al., 2021). In addition, more rapidly migrating and longer mycolic acid methyl esters were found to be present in biofilms compared to planktonic cultures, which was associated with upregulation of *M. abscessus* genes related to mycolic acid chain elongation and desaturation (Dokic et al., 2021). In contrast, another study showed that free mycolates were relatively scarce in the ECM of SCFM-grown biofilms and were similar to those found in planktonic cultures (Belardinelli et al., 2021). The amount of trehalose dimycolate was found to be increased in *M. abscessus* biofilms cultured in SCFM, but not in Middlebrook 7H9 or Hartmans-de Bont broth. In contrast, in another study, no increase in trehalose dimycolate levels in SCFM-grown biofilms was observed, indicating that experimental conditions beyond culture medium influence ECM lipid composition (Hunt-Serracin et al., 2019; Belardinelli et al., 2021). Interestingly, exposure of *M. abscessus* biofilms to *phospholipases* dispersed the SCFM-grown biofilm (Belardinelli et al., 2021), whereas *lipase* exposure did not lead to biofilm dispersal in both SCFM-grown and Sauton’s medium-grown biofilms (Belardinelli et al., 2021; Chakraborty et al., 2021). Biofilms exposed to amikacin or linezolid were shown to contain higher levels of proteins involved in glycerophospholipid metabolism, possibly affecting the lipid composition of the ECM (Rojony et al., 2020).

#### 5.1.3 Carbohydrates

Carbohydrates have been reported to play a role in biofilm adhesion, scaffolding, stability, and immune evasion (Karygianni et al., 2020). In *M. abscessus* biofilms, carbohydrates not only localize with *M. abscessus* itself, but are also present in the inter-bacterial space, where they may exhibit variations in their structure (Belardinelli et al., 2021; Chakraborty et al., 2021; Dokic et al., 2021). The main carbohydrate present in SCFM cultured biofilms was found to be glucose, which is in fact also a component of the SCFM (Belardinelli et al., 2021). Interestingly, arabinose and galactose were substantially more abundant in biofilm ECM compared to extracellular components of planktonic cultures, indicating an important role for these carbohydrates in biofilms, and suggesting an active release from the bacteria (Belardinelli et al., 2021). Supporting this idea, uniquely expressed proteins in *M. abscessus* biofilm included an enzyme involved in arabinan synthesis (Belardinelli et al., 2021). In *M. abscessus* biofilms cultured in Sauton’s medium, cellulose was found to be present (Chakraborty et al., 2021). Exposure of biofilms to an α-mannosidase and cellulose led to a significant dispersal of the biofilm, indicating carbohydrates are indeed a crucial constituent of the *M. abscessus* biofilm (Belardinelli et al., 2021; Chakraborty et al., 2021). In contrast, α-amylase did not lead to biofilm dispersal (Chakraborty et al., 2021).

#### 5.1.4 Proteins

Protein abundance has been reported in *M. abscessus* biofilms, when cultured both in SCFM and Sauton’s medium (Belardinelli et al., 2021; Chakraborty et al., 2021; Dokic et al., 2021). These proteins were found to be localized in the deep layers of the biofilm, with limited bacterial co-localization (Belardinelli et al., 2021; Chakraborty et al., 2021; Dokic et al., 2021). Interestingly, the proteases proteinase K and trypsin did not disperse SCFM established *M. abscessus* biofilms, indicating a less prominent role for proteins in biofilm integrity (Belardinelli et al., 2021). Alternatively, proteins might be important during earlier phases of biofilm maturation, such as the initial attachment stage (Fong and Yildiz, 2015). In contrast, proteinase K exposure led to biofilm dispersal in reductive stress-induced Sauton’s medium *M. abscessus* biofilm, which suggests a potential role in structure and integrity of biofilms in this particular environment (Chakraborty et al., 2021). This again highlights the variability in outcome when studying the ECM of *M. abscessus* biofilms under different environmental conditions. In SCFM cultured biofilms, proteins that are enriched in the ECM are thought to be mainly involved in central carbon metabolism, respiration, amino acid and lipid metabolism, heat shock responses and hypoxia (Belardinelli et al., 2021).

#### 5.1.5 Mechanical properties

The ECM composition has been reported to affect the mechanical properties of the biofilm (Gloag et al., 2020). Studying the mechanical properties of biofilms is important to understand how biofilms respond to chemical and mechanical forces (Gloag et al., 2020). Roughly two different methods to study biofilm mechanics have been distinguished: indentation, which involves forces that are perpendicular to the surface, and shear stress, which involves forces that are parallel to the surface. In general, biofilms have been described as viscoelastic, probably due to adaptations to withstand mechanical stresses from the environment, in contrast to the stiffer nature of planktonic bacteria (Gloag et al., 2020). The viscoelasticity of biofilms is affected by the ratio of bacteria to ECM content and the relative abundance of specific ECM components.

Techniques to study the mechanical properties of biofilms include bulk rheology and microrheology. In bulk rheology, an entire sample is analysed with a rheometer to study the overall mechanical behavior of biofilm, while microrheology is used to study local mechanical responses and thus can investigate heterogeneities in viscoelastic properties within the biofilm matrix. An overview of all techniques to study biofilm rheology is provided by other reviews (Billings et al., 2015; Gordon et al., 2017).

To our knowledge, only one rheology study has been performed on *M. abscessus* biofilms (Gloag et al., 2021). In this study colony biofilms were exposed to uniaxial indentation and shear forces. In alignment with the general consensus of the mechanical properties of biofilms, *M. abscessus* had viscoelastic properties that were similar between smooth and rough morphotypes. In contrast, the force needed to break down the biofilm was greater for rough morphotype biofilms compared to smooth morphotypes and rough morphotypes could be compressed to a further extent under uniaxial indentation forces (Gloag et al., 2021). Studying the mechanical properties of *M. abscessus* biofilms is potentially relevant, as their stiffness is thought to contribute to its relative resistance to airway clearance (Gloag et al., 2021). Furthermore, biofilms might withstand the stress applied by immune cells, hampering phagocytosis (Gordon et al., 2017). The mechanical properties of a biofilm have been linked to its permeability, which is important in the context of drug susceptibility and to understand the accessibility of bacteria in the deeper layers of the biofilm (Gloag et al., 2020). Interestingly, microsensors have been developed to monitor and profile for example oxygen, pH, CO_2_, sulphides, and nitrate throughout the biofilm, providing additional information on the heterogeneity of the biofilm microenvironment (Beyenal and Babauta, 2014).

## 6 Integrating biofilm techniques: targeting the *Mycobacterium abscessus* biofilm

Drug activity testing against *M. abscessus* biofilms can be approached by studying inhibition of biofilm formation or biofilm destruction. In biofilm inhibition studies, planktonic cultures or young biofilms are generally exposed to subinhibitory antibiotic concentrations. After a predefined time period, the impact on biofilm formation is assessed. In the context of biofilm destruction, compounds are added to pre-established biofilms at higher antibiotic concentrations, allowing the study of physical disruption of established structures as well as the killing of bacteria residing within the biofilm. A summary of studies on drug activity against *M. abscessus* biofilms, along with details on the experimental design of the models used is presented in Supplementary Table S1.

### 6.1 Common *Mycobacterium abscessus* drugs

#### 6.1.1 Macrolides: clarithromycin and azithromycin

Although one study showed biofilm inhibition in terms of biofilm biomass upon clarithromycin exposure compared to a no drug control, another study found that clarithromycin did not reduce CFU values in a developing biofilm, indicating that clarithromycin was unable to completely prevent biofilm formation (Flores et al., 2016; Rodríguez-Sevilla et al., 2018b). Clarithromycin, when added to young biofilms cultured for 24 h, arrested mycobacterial growth but was not able to kill *M. abscessus* in these biofilms (Yam et al., 2020; Ganapathy et al., 2021; Negatu et al., 2021; Ganapathy et al., 2023). Studies using 4–5-day old biofilms reported either no or only limited reductions in viable bacteria after clarithromycin exposure, whereas in fully mature biofilms, clarithromycin showed (limited) killing activity, which was suggested to be medium dependent (Greendyke and Byrd, 2008; Ortíz-Pérez et al., 2011; Hunt-Serracin et al., 2019; Lee et al., 2021¸ 2022; Li et al., 2022; Fan et al., 2024). In addition, a reduction in biofilm biomass was reported, suggesting clarithromycin was also able to disrupt the biofilm structure (Hunt-Serracin et al., 2019). Although less studies were performed with azithromycin, the results are in line with those observed for clarithromycin, indicating the prevention of CFU increase in young biofilms without exhibiting bactericidal activity (Clary et al., 2018; Yam et al., 2020). In addition, azithromycin was not able to prevent an increase in viable bacteria within more mature biofilms, and showed minimal killing activity in fully mature biofilms (Belardinelli et al., 2021; Kaur et al., 2024).

#### 6.1.2 Amikacin

In line with the results for the macrolides, amikacin could not fully prevent biofilm development when amikacin was added at the onset of biofilm formation, although it was able to reduce biofilm biomass (Flores et al., 2016; Rodríguez-Sevilla et al., 2018b). In addition, amikacin did not reduce CFU values in single- and dual species biofilm models during biofilm development (Rodríguez-Sevilla et al., 2018b). Importantly, the latter inhibition assay lasted only 6 h, which could have been too short for this purpose. When added to young biofilms (i.e., 24 h) amikacin has been reported to have both inhibitory as well as killing activity (Clary et al., 2018; Rodríguez-Sevilla et al., 2019; Yam et al., 2020). Multiple studies have assessed amikacin’s activity on more mature biofilms, in which different experimental variables were used. In most, but not all, studies, amikacin’s activity was reported to (slightly) reduce the mycobacterial load in biofilms compared to no drug controls (Greendyke and Byrd, 2008; Ortíz-Pérez et al., 2011; Marini et al., 2018; Rodríguez-Sevilla et al., 2019; Paulowski et al., 2022). In fully established biofilms amikacin showed no or limited killing activity and did not reduce biofilm biomass (Flores et al., 2016; Hunt-Serracin et al., 2019; Rojony et al., 2020; Li et al., 2022; Fan et al., 2024; Kaur et al., 2024; Oschmann-Kadenbach et al., 2024).

Interestingly, medium dependent differences in amikacin’s activity against *M. abscessus* biofilms have been observed, with more pronounced killing activity in 7H9 Middlebrook cultured biofilms compared to biofilms cultured in SCFM (Hunt-Serracin et al., 2019). Moreover, biofilm maturity has been observed to affect amikacin’s activity with indications that more mature biofilms have increased resistance to amikacin (Marini et al., 2018; Rodríguez-Sevilla et al., 2019). Another variable reported to affect amikacin’s killing capacity is experimental duration, with longer antibiotic exposure linked to increased activity of amikacin (Paulowski et al., 2022). This is interesting, since the majority of studies expose biofilms for 24 or 48 h to amikacin, and thus potentially do not reach a reliable efficacy assessment. Of note, it remains unclear whether there is a difference in amikacins activity against smooth or rough morphotype biofilms. Only one study addressed this issue but found no difference in amikacin susceptibility between the smooth and rough morphotype in young biofilms (Clary et al., 2018).

#### 6.1.3 Imipenem

Imipenem was reported to inhibit biofilm mass accumulation in developing biofilms (Flores et al., 2016). In addition, imipenem showed inhibitory as well as killing activity in young biofilms (Yam et al., 2020), although these effects were not observed in more mature biofilms (Belardinelli et al., 2021). In fully mature biofilms, imipenem had slight bactericidal activity, but did not reduce biofilm biomass (Flores et al., 2016; Li et al., 2022; Fan et al., 2024).

#### 6.1.4 Cefoxitin

Cefoxitin was demonstrated to have inhibitory as well as killing activity against *M. abscessus* in young biofilms (i.e., 24 h) at concentrations 3 to 4 times higher than the minimal inhibitory concentration in planktonic populations (Yam et al., 2020). While exhibiting bacteriostatic activities against *M. abscessus* within 4-day-old biofilms, this effect was observed only at exceptionally high concentrations (Greendyke and Byrd, 2008). In fully mature biofilms, cefoxitin slightly reduced the mycobacterial load after 48 h of exposure (Hunt-Serracin et al., 2019; Li et al., 2022; Fan et al., 2024).

#### 6.1.5 Tigecycline

Tigecycline showed both inhibitory as well as killing activity against *M. abscessus* in young biofilms (i.e., 24 h) Of note, the killing activity was observed at lower concentrations compared to what was needed for the killing of planktonic *M. abscessus* (Yam et al., 2020). In contrast, only minimal killing activity was observed against *M. abscessus* in fully mature biofilms (Oschmann-Kadenbach et al., 2024).

#### 6.1.6 Linezolid

Linezolid was shown to have inhibitory but not bactericidal activity in young *M. abscessus* biofilms (i.e., 24 h), although requiring a concentration three times higher than needed for the growth inhibition of planktonic *M. abscessus* (Yam et al., 2020). Linezolid did not reduce the number of viable bacteria in fully mature biofilms (Rojony et al., 2020; Yam et al., 2020). Despite this, changes in metabolism were reported, indicating linezolid might impact cellular processes (Rojony et al., 2020).

#### 6.1.7 Clofazimine

Clofazimine showed inhibitory as well as killing activity against young biofilms (i.e., 24 h) (Yam et al., 2020). Interestingly, this killing activity was observed at lower concentrations compared to what was needed for the killing of planktonic *M. abscessus* (Yam et al., 2020). In contrast, clofazimine was not able to prevent growth of *M. abscessus* in 4-day old biofilms, suggesting clofazimine predominantly affects early stage biofilms (Belardinelli et al., 2021).

#### 6.1.8 Others

*Doxycyline* demonstrated inhibitory activity on biofilm formation in terms of biofilm biomass at concentrations lower than the minimal inhibitory concentrations in planktonic bacteria (Flores et al., 2016). However, it only slightly reduced biomass of established biofilms at concentrations exceeding the minimal inhibitory concentration in planktonic *M. abscessus* (Flores et al., 2016). *Minocycline* did neither inhibit biofilm formation, nor kill *M. abscessus* within young biofilms (Yam et al., 2020). Sub-inhibitory *ciprofloxacin* concentrations were able to inhibit biofilm formation in terms of biofilm biomass, but ciprofloxacin did not affect the biomass of pre-established biofilms (Flores et al., 2016; Belardinelli et al., 2021). Conflicting results have been reported on the effect of ciprofloxacin exposure on the mycobacterial load, ranging from total killing to no inhibitory or killing activity (Ortíz-Pérez et al., 2011; Muñoz-Egea et al., 2015; Blanchard et al., 2018; Belardinelli et al., 2021). *Moxifloxacin* was shown to have inhibitory and killing activity against *M. abscessus* within young biofilms (Yam et al., 2020; Ganapathy et al., 2021). One study showed killing activity of moxifloxacin against *M. abscessus* in fully mature biofilms (Kaur et al., 2024). *Rifamycins* have only sporadically been studied in the context of *M. abscessus* biofilm. *Rifampicin* did not affect *M. abscessus* in 3-day old biofilms, whereas *rifabutin* reduced CFU values in 3-day old biofilms in a time dependent manner, achieving a 3-log_10_ reduction in percentage of viable bacteria after 96 h of exposure (Paulowski et al., 2022). In line, rifabutin was able to reduce CFU values and colony volume in a colony biofilm (Aguilera-Correa et al., 2023). Notably, the reduction in CFU counts was greater in rough morphotype *M. abscessus* compared to the smooth mophotype (Aguilera-Correa et al., 2023). The sulfanomide *sulfamethoxazole* was able to inhibit biofilm formation at sub-inhibitory concentrations but was not able to destruct established biofilms (Flores et al., 2016). Only limited studies have been conducted on *bedaquiline’s* activity against *M. abscessus* biofilm. Bedaquiline did not inhibit biofilm formation when administered at the onset of biofilm formation. Yet, it reduced CFU values in smooth—but not in rough—morphotypes in a 2-day old colony biofilm (Chakraborty et al., 2021; Aguilera-Correa et al., 2023). *Ceftazidime* and *colistin* are not commonly used for treatment of nontuberculous mycobacteria but are more common in the treatment of *P. aeruginosa* infections. Therefore, ceftazidime and colistin were tested in a dual-species biofilm with *P. aeruginosa*. Indeed, ceftazidime and colistin did not reduce CFU values of *M. abscessus* in single- and dual-biofilm (Rodríguez-Sevilla et al., 2018b, 2019). However, selectively targeting *P. aeruginosa* in a dual-species biofilm could lead to increased survival and thus a competitive advantage of *M. abscessus* (Rodríguez-Sevilla et al., 2019).

### 6.2 Novel compounds

Several novel compounds, with diverse modes of action, have been tested against *M. abscessus* biofilms under various experimental conditions (Supplementary Table S2). Notably, these compounds have only been studied once in this context, highlighting the need to further validate their therapeutic potential. EC/11770, EC/11716, IMA6 and SA23, targeting protein synthesis, DNA replication, mycolic acid transport and the electron transport chain, respectively, exhibited both inhibitory as well as killing activity against *M. abscessus* within young biofilms (i.e., 24 h) (Yam et al., 2020; Ganapathy et al., 2021, 2023). In addition, essential oils derived from *Cymbopogon Flexuos* species and the essential oil constituent carvacrol reduced biofilm biomass of developing as well as pre-established biofilms (Rossi et al., 2017; Marini et al., 2019). The aminoimidazole AB-2–29, several sulfonamides complexed with metals, and several inhibitors of the dormancy survival regulon DosRS (which is essential for *M. abscessus* to persist under hypoxic conditions) inhibited biofilm biomass during biofilm development, but did not reduce the biomass of pre-established biofilms (Bonez et al., 2021; Belardinelli et al., 2022a,b). Moreover, multiple 2-aminoimidazoles, the antibacterial protein RP557, *Panax quinquefolius*, *Coptic chinensis* and its main constituent berberine were able to inhibit biofilm development in terms of biomass, yet their activity on pre-established biofilms was not assessed (Tseng et al., 2020; Li et al., 2022; Belardinelli et al., 2022a; He et al., 2023). For RP557 and *Panax quinquefolius* bacterial inhibition in terms of mycobacterial load and metabolic activity was observed as well. Moreover, RP557 enhanced the killing activity of clarithromycin, amikacin, imipenem and cefoxitin in fully mature biofilms (Li et al., 2022). Clomiphene citrate, which is hypothesized to disrupt the mycobacterial membrane, and 10-DEBC, an inhibitor of the Akt pathway, inhibited the growth of 5-days old biofilms during a 4-day exposure (total assay duration of 9 days), but their killing activity was not assessed (Lee et al., 2021, 2022). 5-aminovulinic acid—photodynamic therapy, HuTipMab (targeting DNA binding proteins) and curcumin reduced biomass in established biofilms (Marini et al., 2018; Yue et al., 2022; Kurbatfinski et al., 2023). In addition, curcumin enhanced the biofilm destructing capacity of amikacin in 4- and 8-day old biofilms (Marini et al., 2018). Colloidal silver, the dendritic amphiphile 3Cam19, the thiopeptide antibiotic NF1001 and the rifamycin derivate 5j reduced mycobacterial viability in established biofilms (Falkinham et al., 2012; Paulowski et al., 2022; Feizi et al., 2023; Kaur et al., 2024). Finally, the antimicrobial peptide D-hLF-1–11, a derivate of lactoferrin, seemed to not affect *M. abscessus* biofilm biomass (Intorasoot et al., 2022).

## 7 Concluding remarks

*M. abscessus* biofilm formation is a complex process, which might contribute to the high treatment failure rates observed in clinical settings. Standard preclinical drug activity assays focus on planktonic bacteria, leaving the response of *M. abscessus* within biofilms relatively unexplored. This might contribute to the lack of correlation between *in vitro* susceptibility and clinical performance of the drug. Setting up biofilm experiments can be challenging given the number of models and variables that might affect biofilm characteristics and its susceptibility to antibiotics. The choice of *M. abscessus* strain, culture medium, and biofilm age affect important biofilm characteristics such as its macroscopic structure, ECM composition, and/or physiology. However, in most cases the exact influence of different experimental variables remains unclear, adding complexity to the experimental set-up and interpretation of results. As such, absence of experimental details in published studies can hinder the reproducibility and interpretability of studies. Despite the challenges associated with technical variations, *M. abscessus* consistently forms biofilm under these various conditions, highlighting the significance of this trait. Beyond selecting appropriate laboratory models for biofilm culture (i.e., models with translational value), adopting a multi-technique approach to study biofilms is crucial for thorough and accurate analysis, as each individual technique has its own analytical perspectives and limitations.

As to drug activity assessment, there is only limited data available on the activity of traditional antibiotics against *M. abscessus* biofilms, with indications that traditional drugs are often not as active against *M. abscessus* residing in biofilm compared to planktonic *M. abscessus*. Thus, including *M. abscessus* biofilm models for comprehensive preclinical drug activity testing could be important to improve the translational value of *in vitro* drug development as well as in order to select the optimal combination of drugs to target both planktonic mycobacteria and those residing in biofilms. However, for proper assessment of the data gained from these biofilm models, certain experimental aspects should be considered. Careful consideration of adequate readouts is important in interpreting data on drug activity. For example, CFU determination and crystal violet staining are the most commonly used methods for biofilm quantification, each providing information on other parameters of the biofilm: viability and biomass, respectively. Using both techniques simultaneously contribute to the understanding whether a compound affects the biofilm structure or the bacteria itself, which is important to try and select the optimal combination of antibiotics against infections with this difficult-to-treat and biofilm forming *M. abscessus.* In addition to using different read-outs, careful consideration of culture medium, exposure time, and biofilm age is needed. Early-stage biofilms are often more susceptible to antibiotics, and longer drug exposure times might lead to more drug activity in biofilms. In most studies, the exposure time was 24 to 48 h only, while prolonged exposure times might have led to more pronounced biofilm destruction (Paulowski et al., 2022). Moreover, differences in biofilm characteristics between the smooth and rough colony morphotype in *M. abscessus* is an underexplored topic which could be an important variable to take into account, especially since differences in morphotype have been linked to clinical outcome (Sanguinetti et al., 2001; Catherinot et al., 2009).

The lack of standardized experimental protocols complicates comparisons between studies. However, selecting the one ideal model for *M. abscessus* biofilm research would be very challenging. In fact, the use of different models offers valuable insights into diverse environments, providing a comprehensive understanding of biofilm characterization and behavior as well as drug activity. It is important to recognize that *M. abscessus* encounters complex environments in patients. Various optimizations have therefore already been implemented into laboratory models, including the use of artificial sputum medium and the creation of dual-species biofilms. Careful consideration of the *M. abscessus* environment in patients and aligning this with a specific research question is crucial to select a model that balances complexity, reproducibility and pragmatism (Coenye et al., 2020).

In conclusion, selected technical tools and variables frame the way we interpret the *M. abscessus* biofilm. In this framing process, we struggle with the challenge of distinguishing between the inherent nature of the biofilm and the impact of our observational tools and selected environmental conditions. Understanding the frame that we use is important in understanding the complex nature of *M. abscessus* biofilm and its behavior upon antibiotic exposure. Moreover, it helps to bridge the gap between *in vitro* models and clinical outcome, thereby increasing the preclinical value of our research.

## Author contributions

HM: Writing – original draft, Conceptualization, Writing – review & editing. SM: Conceptualization, Supervision, Writing – review & editing. NA: Writing – review & editing. JS: Writing – review & editing. HB: Conceptualization, Supervision, Writing – review & editing.
