# Supplementary material for: A laboratory perspective on Mycobacterium abscessus biofilm culture, characterization and drug activity testing
Source: Front Microbiol. 2024 Apr 16;15:1392606. doi: 10.3389/fmicb.2024.1392606 (PMC11058659; doi:10.3389/fmicb.2024.1392606)
Supplement: Supplementary file 1 [file Table_1.DOCX]

Supplementary Material

# Literature search strategy

## Search terms used in databases

*Embase*

('Mycobacterium abscessus'/exp OR (((Mycobacter* OR M) NEAR/3 (abscessus OR massiliens* OR boletti))):ab,ti,kw) **AND** (biofilm/exp OR 'antibiofilm activity'/de OR (biofilm* OR bio-film* OR antibiofilm*):ab,ti,kw) NOT (review/exp OR review:ti) NOT ([Conference Abstract]/lim OR [Conference Review]/lim)

Medline

(Mycobacterium abscessus/ OR (((Mycobacter* OR M) ADJ3 (abscessus OR massiliens* OR boletti))).ab,ti,kf.) **AND** (exp Biofilms/ OR (biofilm* OR bio-film* OR antibiofilm*).ab,ti,kf.) NOT (Review/ OR review.ti.) NOT (congres* OR abstract*).pt.

Web of Science

TS=(((((Mycobacter* OR M) NEAR/2 (abscessus OR massiliens* OR boletti)))) **AND** ((biofilm* OR bio-film* OR antibiofilm*))) NOT TI=review NOT DT=(Meeting Abstract OR Meeting Summary)

Google Scholar

"Mycobacterium|M abscessus" biofilm|antibiofilm|"bio film" -review -infection

'Mycobacterium|M abscessus' biofilm|antibiofilm|'bio film' -review –infection

## Number of records on 20-02-2024

| **Database searched** | **Platform** | **Years of coverage** | **Records** | **Records after duplicates removed** |
| --- | --- | --- | --- | --- |
| Medline ALL | Ovid | 1946 - Present | 106 | 104 |
| Embase | Embase.com | 1971 - Present | 127 | 40 |
| Web of Science Core Collection* | Web of Knowledge | 1975 - Present | 132 | 35 |
| Additional Search Engines: Google Scholar** (50 top-ranked) | | | 50 | 45 |
| **Total** | | | **415** | **224** |

*Science Citation Index Expanded (1975-present) ; Social Sciences Citation Index (1975-present) ; Arts & Humanities Citation Index (1975-present) ; Conference Proceedings Citation Index- Science (1990-present) ; Conference Proceedings Citation Index- Social Science & Humanities (1990-present) ; Emerging Sources Citation Index (2005-present)

**Google Scholar was searched via "Publish or Perish" to download the results in EndNote.

No other database limits were used than those specified in the search strategies

# Supplementary Table 1

Overview of drug activity of existing drugs against *M. abscessus* biofilm. Biofilm maturity = biofilm age before compound exposure. * = agar based medium. CFU = Colony Forming Units; RFI = Relative Fluorescence Intensity; RPMI = Roswell Park Memorial Institute; FBS = fetal bovine serum; MB = Middlebrook; MIC = minimal inhibitory concentration; MBC = minimal bactericidal concentration; OADC = Oleic Albumin Dextrose Catalase; OD = optical density; SCFM = synthetic cystic fibrosis medium; HBSS = Hank’s balanced salt solution; HdB = Hartmans-de Bont; RFI = relative fluorescence intensity; MBEC = minimum biofilm eradication concentration; IC50 = half-maximal inhibitory concentration; ADC = albumin, dextrose, catalase; AMK = amikacin; CZA = ceftazidime; IMP = imipenem; CLR = clarithromycin.

| Concentration | Biofilm maturity | Exposure Time | Culture medium | Technique | Activity | | Reference |
| --- | --- | --- | --- | --- | --- | --- | --- |
| Amikacin | | | | | | | |
| 17 µg/mL | 0 hours | 6 hours | GTSF-2 medium + supplements | CFU determination | No reduction in CFU values compared to no drug control | | Rodríguez-Seville et al., 2019 |
| 0.625 – 2 µg/mL | 0 hours | 7 days | 7H9 MB | Crystal Violet staining | 0.5-2 µg/mL amikacin reduced OD value with ±0.3 – 1.5 respectively compared to no drug control | | da Costa Flores et al., 2016 |
| 0 – 256 µg/mL | 24 hours | 24 hours | RPMI + 10% FBS | CFU determination, Relative Fluorescence Intensity (RFI) | < 1 log_10_ CFU reduction and no RFI reduction in smooth and rough biofilms compared to no drug control | | Clary et al., 2018 |
| 0 – 100 µM | 24 hours | 48 hours | 7H9 MB without Tween 80 | CFU determination | MIC: 0.5 µM (biofilm) and 2 µM (planktonic)  MBC: 25 µM (biofilm) and 12.5 µM (planktonic) | | Yam et al., 2020 |
| 80 µg/mL | 24 – 72 hours | 24 hours | Tryptic soy agar + 5% sheep blood* | CFU determination | Reduction of ±4 and ±0.5 Log_10_ CFU/cm^2^ in MAB *single*-species biofilms at 24h and 72h, respectively, compared to no drug control; reduction in CFU values of ±1-2.5 and ≤ 0.5 Log_10_ CFU/cm^2^ in *dual*-species biofilms at 24h and 72h, respectively, compared to no drug control | | Rodríguez-Seville et al., 2018b |
| 10 µg/mL | 3 days | 4 days | Mueller-Hinton II | CFU determination | Time-dependent reduction of % viable cells, with 0.1% viable cells after 96-hours of exposure compared to start of exposure | | Paulowski et al., 2022 |
| 32 – 128 µg/mL | 4-8 days | 24 hours | 7H9 MB + OADC and glycerol | Safranin staining | 77-100% reduction in OD values at day 4 and 8 compared to no drug control when exposed to amikacin alone; enhanced killing activity of amikacin when exposed in combination with curcumin | | Marini et al., 2018 |
| 0 – 256 µg/mL | 4 days | 24 hours | SCFM | Resazurine staining | MIC: > 256 µg/mL (biofilm) and 16 µg/mL (planktonic) | | Belardinelli et al., 2021 |
| 0 – 512 µg/mL | 4 days | 24 hours | Sauton’s medium | CFU determination | Reduction up to 1.1 log_10_ units compared to no drug control, maximal activity achieved at 64 µg/mL | | Greendyke & Bird, 2008 |
| 50 µg/mL | 5 days | 24 hours | Tryptic soy agar + 5% sheep blood* | CFU determination | % of viable bacteria is 40 – 67% after exposure | | Ortíz-Pérez et al., 2011 |
| 0 – 100 µg/mL | 6 days | 24-48 hours | 7H9 MB, HdB, SCFM | CFU determination | ±1-2, 1 and <1 log_10_ reduction at 100 µg/mL in 7H9 MB, HdB, SCFM, respectively, compared to no drug control | | Hunt-Serracin et al., 2019 |
| 8 – 256 µg/mL | 7 days | 48 hours | Sauton’s medium | CFU determination | <1 log_10_ reduction in CFU values compared to start of exposure when exposed to amikacin alone; amikacin in combination with RP557 reduced CFU values with ±1-2 log_10_ at 256 µg/mL | | Li et al., 2022 |
| 2 – 128 µg/mL | 7 days | 24 hours | 7H9 MB | Crystal Violet staining | No reduction in OD value compared to no drug control | | da Costa Flores et al., 2016 |
| 32 µg/mL | 7 days | 24 hours - 6 days | HBSS | CFU determination, proteomics | No reduction in CFU values compared to no drug control, differentially expressed proteins | | Rojony et al., 2020 |
| 0 – 128 µg/mL | 7 days | 5 days | 7H9 + 10% OADC | CFU determination | <1 log_10_ reduction in CFU values compared to no drug control | | Schmann-Kadenbach et al., 2024 |
| 16 – 256 µg/mL | 7 days | 7 days | Sauton’s medium + 10% ADC | CFU determination | Concentration dependent reduction in CFU value (maximal activity: < 2 log_10_ reduction) | | Kaur et al., 2023 |
| 0 – 128 µg/mL | 14 days | 48 hours | Sauton’s medium | CFU determination | Concentration dependent reduction in CFU values (<1 log_10_ reduction at 128 µg/mL compared to no drug control) | | Fan et al., 2024 |
| Azithromycin | | | | | | | |
| 0-256 µg/mL | 24 hours | 24 hours | RPMI + 10% FBS | CFU determination, Relative Fluorescence Intensity (RFI) | No CFU and RFI reduction in smooth and rough biofilms compared to no drug control | | Clary et al., 2018 |
| 0 – 100 µM | 24 hours | 48 hours | 7H9 MB without Tween 80 | CFU determination | MIC: 2 µg/mL (biofilm and planktonic)  MBC: > 100 µM (biofilm and planktonic) | | Yam et al., 2020 |
| 0 – 256 µg/mL | 4 days | 24 hours | SCFM | Resazurine staining | MIC: > 256 µg/mL (biofilm) and 2 µg/mL (planktonic) | | Belardinelli et al., 2021 |
| 16 – 256 µg/mL | 7 days | 7 days | Sauton’s medium + 10% ADC | CFU determination | Concentration dependent reduction in CFU values (maximal activity: < 0.5 log_10_ reduction) | | Kaur et al., 2023 |
| Bedaquiline | | | | | | | |
| 1 – 100x MIC | 0 hours | 29 hours | Sauton’s medium | CFU determination | No reduction in CFU values compared to no drug control | | Chakraborty et al., 2021 |
| 1 µg/mL | 48 hours | 3 days | 7H10 MB* | CFU determination,  colony volume | Reduction in CFU value (29%) and colony volume (27.5%) for smooth morphotype, only reduction in colony volume (28%) only for rough morphotype compared to a no drug control | | Aguilera-Correa et al., 2023 |
| Cefoxitin | | | | | | | |
| 0 – 100 µM | 24 hours | 48 hours | 7H9 MB without Tween 80 | CFU determination | MIC: 50 µM (biofilm) and 16 µM (planktonic)  MBC: 100 µM (biofilm) and 25 µM (planktonic) | | Yam et al., 2020 |
| 0 – 1024 µg/mL | 4 days | 24 hours | Sauton’s medium | CFU determination | Only bacteriostatic activity at 1024 µg/mL | | Greendyke & Byrd, 2008 |
| 0 – 256 µg/mL | 4 days | 24 hours | SCFM | Resazurine staining | MIC: > 256 µg/mL (biofilm) and 16 µg/mL (planktonic) | | Belardinelli et al., 2021 |
| 0 – 250 µg/mL | 6 days | 24-48 hours | 7H9 MB, HdB, SCFM | CFU determination | < 1 log_10_ reduction in 7H9 MB, HdB and SCFM compared to no drug control | | Hunt-Serracin et al., 2019 |
| 8 – 256 µg/mL | 7 days | 48 hours | Sauton’s medium | CFU determination | ±1 log_10_ reduction compared to start of exposure, with maximal activity reached at 32 µg/mL; cefoxitin in combination with RP557 reduced CFU values with up to ±1 log_10_ at 256 µg/mL | | Li et al., 2022 |
| 0 – 128 µg/mL | 14 days | 48 hours | Sauton’s medium | CFU determination | Concentration dependent reduction in CFU values (±1 log_10_ reduction at 128 µg/mL compared to no drug control) | | Fan et al., 2024 |
| Ciprofloxacin | | | | | | | |
| 0.31 – 1 µg/mL | 0 hours | 7 days | 7H9 MB | Crystal Violet staining | 0.625-1 µg/mL reduced OD value with ±0.2-1.2, respectively, compared to no drug control | | Da Costa Flores et al., 2016 |
| 0.125 – 16 µg/mL | 1-4 days | 1-3 days | 7H9 MB, Mueller-Hinton broth | MBEC assay, CLSM | MBEC of 1024 µg/mL; reduction in biofilm thickness | | Muñoz-Egea et al., 2015 |
| 0 – 256 µg/mL | 4 days | 24 hours | SCFM | Resazurine staining | MIC: > 256 µg/mL (biofilm) and 8 µg/mL (planktonic) | | Belardinelli et al., 2021 |
| 50 µg/mL | 5 days | 24 hours | Tryptic soy agar + 5% sheep blood* | CFU determination | % of viable bacteria is 67 – 82% after exposure | | Ortíz-Pérez et al., 2011 |
| 0.31 – 32 µg/mL | 7 days | 24 hours | 7H9 MB | Crystal Violet staining | No reduction in OD value compared to no drug control | | Da Costa Flores et al., 2016 |
| 100 µg/mL | 7 days | 4-7 days | HBSS | CFU determination | 58% reduction in CFU values compared to no drug control | | Blanchard et al., 2018 |
| Clarithromcyin | | | | | | | |
| 1.1 µg/mL | 0 hours | 6 hours | GTSF-2 medium + supplements | CFU determination | No reduction in CFU values compared to no drug control | | Rodríguez-Seville et al., 2019 |
| 0.5 – 16 µg/mL | 0 hours | 7 days | 7H9 MB | Crystal Violet staining | 2-16 µg/mL clarithromycin reduced OD value with ±0.2-1.2, respectively, compared to no drug control | | da Costa Flores et al., 2016 |
| 2 – 16 µM | 24 hours | 48 – 72 hours | 7H9 MB without Tween 80 | CFU determination | No reduction in CFU values compared to start of exposure | | Negatu et al., 2021 |
| 0 – 100 µM | 24 hours | 48 hours | 7H9 MB without Tween 80 | CFU determination | MIC: 3 µM (biofilm) and 0.2 µM (planktonic)  MBC: > 100 µM (biofilm and planktonic) | | Yam et al., 2020 |
| 0.195 – 100 µM | 24 hours | 48 hours | 7H9 MB without Tween 80 | CFU determination | MIC: 1.6 µM (biofilm) and 0.28 µM (planktonic)  MBC: > 100 µM (biofilm and planktonic) | | Ganapathy et al., 2021 |
| 0.195 – 100 µM | 24 hours | 48 hours | 7H9 MB without Tween 80 | CFU determination | MIC: 1.6 µM (biofilm) and 0.28 µM (planktonic)  MBC: > 100 µM (biofilm and planktonic) | | Ganapathy et al., 2021 (2) |
| 0 – 512 µg/mL | 4 days | 24 hours | Sauton’s medium | CFU determination | Reduction of 0.6 log_10_ CFU values compared to no drug control, maximal activity achieved at 64 µg/mL | | Greendyke & Byrd, 2008 |
| 0.01 – 100 µg/mL | 5 days | 4 days | Unclear | Resazurine staining | IC_50_ > 100 µg/mL in biofilm compared to 0.046 µg/mL in planktonic bacteria | | Lee et al., 2021 |
| 50 µg/mL | 5 days | 24 hours | Tryptic soy agar + 5% sheep blood* | CFU determination | % of viable bacteria is 68 – 100% after exposure | | Ortíz-Pérez et al., 2011 |
| 1 – 200 µg/mL | 5 days | 4 days | Unclear | Resazurine staining | IC_50_ > 100 µg/mL in biofilm compared to 0.034 µg/mL in planktonic bacteria | | Lee et al., 2022 |
| 0 – 300 µg/mL | 6 days | 24-48 hours | 7H9 MB, HdB, SCFM | CFU determination | < 1 log_10_ reduction in 7H9 MB and HdB compared to no drug control, ±1 log_10_ reduction in SCFM | | Hunt-Serracin et al., 2019 |
| 16 - 512 µg/mL | 7 days | 24 hours | 7H9 MB | Crystal Violet staining | 16-512 µg/mL reduced OD value with ±0.3 compared to no drug control | | da Costa Flores et al., 2016 |
| 8 – 256 µg/mL | 7 days | 48 hours | Sauton’s medium | CFU determination | < 1 log_10_ reduction compared to start of exposure when exposed to clarithromycin alone. Clarithromycin in combination with RP557 reduced CFU values with up to ±1.5 log_10_ at 256 µg/mL | | Li et al., 2022 |
| 0 – 128 µg/mL | 14 days | 48 hours | Sauton’s medium | CFU determination | Concentration dependent reduction in CFU values (<1 log_10_ reduction at 128 µg/mL compared to no drug control) | | Fan et al., 2024 |
| Clofazimine | | | | | | | |
| 0 – 100 µM | 24 hours | 48 hours | 7H9 MB without Tween 80 | CFU determination | MIC: 9 µM (biofilm) and 2 µM (planktonic)  MBC 12.5 µM (biofilm) and > 100 µM (planktonic) | | Yam et al., 2020 |
| 0 – 256 µg/mL | 4 days | 24 hours | SCFM | Resazurine staining | MIC: > 256 µg/mL (biofilm) and 0.5 µg/mL (planktonic) | | Belardinelli et al., 2021 |
| Colistin | | | | | | | |
| 2 µg/mL | 0 hours | 6 hours | GTSF-2 medium + supplements | CFU determination | No reduction in CFU values compared to no drug control | | Rodríguez-Seville et al., 2019 2 |
| 80 µg/mL | 24 – 72 hours | 24 hours | Tryptic soy agar + 5% sheep blood* | CFU determination | No reduction in CFU values compared to no drug control | | Rodríguez-Seville et al., 2019 |
| Doxycycline | | | | | | | |
| 0.04 – 0.125 µg/mL | 0 hours | 7 days | 7H9 MB | Crystal Violet staining | 0.016-125 µg/mL reduced OD value with ±0.2-1.3, respectively, compared to no drug control | | Da Costa Flores et al., 2016 |
| 0.125 – 4 µg/mL | 7 days | 24 hours | 7H9 MB | Crystal Violet staining | 2-4 µg/mL reduced OD value with ±0.2 compared to no drug control | | Da Costa-Flores et al., 2016 |
| Imipenem | | | | | | | |
| 0.06 – 2 µg/mL | 0 hours | 7 days | 7H9 MB | Crystal Violet staining | 2 µg/mL reduced OD value with ±1.5 compared to no drug control | | Da Costa Flores et al., 2016 |
| 0 – 100 µM | 24 hours | 48 hours | 7H9 MB without Tween 80 | CFU determination | MIC: 20 µM (biofilm) and 5 µM (planktonic)  MBC 50 µM (biofilm) and 12.5 µM (planktonic) | | Yam et al., 2020 |
| 0 – 256 µg/mL | 4 days | 24 hours | SCFM | Resazurine staining | MIC: > 256 µg/mL (biofilm) and 8 µg/mL (planktonic) | | Belardinelli et al., 2021 |
| 2 – 64 µg/mL | 7 days | 24 hours | 7H9 MB | Crystal Violet staining | No reduction in OD value compared to no drug control | | Da Costa-Flores et al., 2016 |
| 8 – 256 µg/mL | 7 days | 48 hours | Sauton’s medium | CFU determination | ±1 log_10_ reduction compared to start of exposure when exposed to imipenem alone, with maximal activity achieved at 64 µg/mL. Imipenem in combination with RP557 reduced CFU values with up to ±1.5 log_10_ at 256 µg/mL | | Li et al., 2022 |
| 0 – 128 µg/mL | 14 days | 48 hours | Sauton’s medium | CFU determination | Concentration dependent reduction CFU values (±1 log_10_ reduction at 128 µg/mL compared to no drug control) | | Fan et al., 2024 |
| Linezolid | | | | | | | |
| 0 – 100 µM | 24 hours | 48 hours | 7H9 MB without Tween 80 | CFU determination | MIC: 12.5 µM (biofilm) and 4 µM (planktonic);  MBC: > 100 µM (biofilm and planktonic) | | Yam et al., 2020 |
| 32 µg/mL | 7 days | 24 hours - 6 days | HBSS | CFU determination, proteomics | No reduction in CFU values compared to no drug control, differentially expressed proteins. | | Rojony et al., 2020 |
| Minocycline | | | | | | | |
| 0 – 100 µM | 24 hours | 48 hours | 7H9 MB without Tween 80 | CFU determination | MIC: > 100 µM (biofilm) and 31 µM (planktonic)  MBC: > 100 µM (biofilm and planktonic) | | Yam et al., 2020 |
| Moxifloxacin | | | | | | | |
| 0 – 100 µM | 24 hours | 48 hours | 7H9 MB without Tween 80 | CFU determination | MIC:6 µM (biofilm) and 2 µM (planktonic)  MBC: 25 µM (biofilm) and 3 µM (planktonic) | | Yam et al., 2020 |
| 0.195 – 100 µM | 24 hours | 48 hours | 7H9 MB without Tween 80 | CFU determination | MIC: 6 µM (biofilm) and 1.9 µM (planktonic)  MBC: 25 µM (biofilm) and 3.1 µM (planktonic) | Ganapathy et al., 2021 | |
| 4 – 64 µg/mL | 7 days | 7 days | Sauton’s medium + 10% ADC | CFU determination | Concentration dependent reduction in CFU values (maximal activity (< 1.5 log_10_ reduction at 64 µg/mL compared to no drug control) | Kaur et al., 2023 | |
| Tigecycline | | | | | | | |
| 0 – 100 µM | 24 hours | 48 hours | 7H9 MB without Tween 80 | CFU determination | MIC: 9 µM (biofilm) and 2 µM (planktonic)  MBC: 75 µM (biofilm) and 100 µM (planktonic) | | Yam et al., 2020 |
| 0 – 8 µg/mL | 7 days | 5 days | 7H9 + 10% OADC | CFU determination | <1 log_10_ reduction in CFU values compared to no drug control | | Schmann-Kadenbach et al., 2024 |
| Sulfamethoxazole | | | | | | | |
| 0.25 – 8 µg/mL | 0 hours | 7 days | 7H9 MB | Crystal Violet staining | 1-8 µg/mL reduced OD value with ±0.1-1.3, respectively, compared to no drug control | | Da Costa-Flores et al., 2016 |
| 8 – 256 µg/mL | 7 days | 24 hours | 7H9 MB | Crystal Violet staining | No reduction in OD value compared to no drug control | | Da Costa-Flores et al., 2016 |
| Rifabutin | | | | | | | |
| 1 µg/mL | 48 hours | 3 days | 7H10 MB* | CFU determination, colony volume | Reduction in CFU value (29%) and colony volume (27.5%) for smooth morphotype, only reduction in colony volume (28%) only for rough morphotype compared to no drug control | | Aguilera-Correa et al., 2023 |
| 10 µg/mL | 3 days | 4 days | Mueller-Hinton II | CFU determination | Time-dependent reduction of % viable cells, with 0.1% viable cells after 96-hours of exposure compared to start of exposure | | Paulowski et al., 2022 |
| Rifampicin | | | | | | | |
| 10 µg/mL | 3 days | 4 days | Mueller-Hinton II | CFU determination | No reduction in CFU values compared to the start of exposure | | Paulowski et al., 2022 |
| Amikacin + Ceftazidime | | | | | | | |
| 80 µg/mL (AMK) 80 µg/mL (CZA) | 24 – 72 hours | 24 hours | Tryptic soy agar + 5% sheep blood* | CFU determination | Reduction of ±4 and ± 0.6 Log_10_ CFU/cm^2^ in MAB *single*-species biofilms at 24h and 72h, respectively, compared to no drug control; reduction in CFU values of ± 1-2 and ≥ 0.5 Log_10_ CFU/cm^2^ in *dual*-species biofilms at 24h and 72h, respectively, compared to no drug control | | Rodríguez-Seville et al., 2019 |
| Imipenem + Clarithromycin | | | | | | | |
| 15 µg/mL (IMP) + 20 µg/mL (CLR) | 3-11 days | 48 hours | SCFM | CFU determination | < 1 log_10_ reduction compared to start of exposure | | Hunt-Serracin et al., 2019 |

# Supplementary Table 2

Drug activity testing of novel compounds against *M. abscessus* biofilms. “mechanism of action”: stated according to the description provided in the corresponding article. CFU = Colony Forming Units; IC50 = half-maximal inhibitory concentration; MIC = minimal inhibitory concentration; MBC = minimal bactericidal concentration; OD = optical density; 10-DEBC = 10-4'-(N,N-diethylamino)butyl-2-chlorophenoxazine hydrochloride; IMA6 = N-substituted indolemethylamine; SA23 = N1,N3-dialkyl substituted dioxonaphthoimidazolium; XTT = Tetrazolium chloride

| Compound | Mechanism of action | Technique | Activity | Reference |
| --- | --- | --- | --- | --- |
| 10-DEBC hydrochloride | Selective Akt inhibitor | Resazurine staining | IC_50_: 38.72 µg/mL (biofilm) and 3.01-5.81 µg/mL (planktonic)  MIC_90_: 50 µg/mL (biofilm) and 4.48-9.53 µg/mL (planktonic) | Lee et al., 2022 |
| 5j | Rifamycin derivate | CFU determination | Time-dependent reduction of % viable cells, with 0.1% viable cells after 96-hours of exposure compared to start of exposure | Paulowski et al., 2022 |
| 2-aminoimidazoles | Targeting two-component signaling systems, targeting membrane bioenergetics, membrane permeabilization | Crystal Violet staining | 12 out of 30 compounds inhibited biofilm formation with IC_50_ values 2- to 4-fold below their measured MIC | Belardinelli et al., 2022 |
| AB-2-29 | Zinc chelator, precise mechanism of action unknown | Crystal Violet staining | Inhibition assay: IC_50_ of 6.25 – 50 µg/mL; concentration dependent reduction in OD value, with a maximal reduction of > 0.5; destruction assay: no reduction in OD value | Belardinelli et al., 2022 |
| EC/11770 | Leucyl-tRNA synthetase inhibitor | CFU determination | MIC: 3.1 µM (biofilm) and 3.0 µM (planktonic)  MBC: 50 µM (biofilm) and > 100 µM (planktonic) | Ganapathy et al., 2023 |
| EC/11716 | DNA gyrase inhibitor | CFU determination | MIC: 0.78 µM (biofilm) and 1.5 µM (planktonic)  MBC: 6.3 µM (biofilm) and 3.1 µM (planktonic) | Ganapathy et al., 2021 |
| 3CAm19 | Perturbation of membrane processes | CFU determination | Survival fraction of 0.050 after 3 hour exposure compared to the start of exposure. | Falkinham III et al., 2012 |
| RP557 | Hypothesized to downregulate nitrogen, fatty acid, and peptide anabolic pathways. | CFU determination, Crystal Violet Staining, microscopy (live/dead staining) | ± 0.5 reduction in OD value; thinner and looser biofilm; enhancement dead bacteria staining signal; enhancement of amikacin, clarithromycin, imipenem, and cefoxitin activity. | Li et al., 2021 |
| Clomiphene citrate | Hypothesized to disrupt mycobacterial membrane and inhibit cell wall biosynthesis | Resazurine staining | IC_50_: 15.94 µg/mL (biofilm) and 4.29 µg/mL (planktonic) | Lee et al., 2021 |
| Colloidal silver | Disruption cell wall, ROS production | Resazurine staining, microscopy (live/dead staining) | Biofilm eradication percentage > 50%, concentration dependent increase in dead bacteria staining signal | Feizi et al., 2023 |
| IMA6 | Hypothesized to inhibit mycolic acid transport | CFU determination | MIC: 6 µM (biofilm) and 3 µM (planktonic)  MBC: 75 µM (biofilm) and 3 µM (planktonic) | Yam et al., 2020 |
| SA23 | Affects electron transport chain | CFU determination | MIC: 75 µM (biofilm) and 3 µM (planktonic)  MBC: 100 µM (biofilm) and 25 µM (planktonic) | Yam et al., 2020 |
| D-Hlf-1-11 | Unknown | Crystal Violet staining | No reduction in relative biomass | Intorasoot et al., 2022 |
| 5-aminovulinic acid - photodynamic therapy | Reactive oxygen species production, disruption protein synthesis | CFU determination, Crystal Violet staining, microscopy (live/dead staining) | 52-64% and 23-45% reduction in CFU and OD values, respectively; disruption biofilm structure, increase in dead bacteria staining signal compared to unexposed control | Wang et al., 2022 |
| Sulfonamide complexed with metals | Folate biosynthesis inhibitor; inhibition c-di-GMP | Crystal Violet staining | Inhibition assay: reduction in OD value compared to unexposed control  Destruction assay: no reduction in OD value compared to unexposed control | Bonez et al., 2021 |
| MSU-39446 | DosRST inhibitor | Crystal Violet staining, microscopy | Inhibition assay: reduction in OD value compared to unexposed control  Destruction assay: no reduction in OD value compared to unexposed control | Belardinelli et al., 2022 |
| Artemisin | DosRST inhibitor | Crystal Violet staining, microscopy | Inhibition assay: concentration dependent reduction in OD value; decrease in biofilm thickness and biofilm density  Destruction assay: no reduction in OD value compared to unexposed control | Belardinelli et al., 2022 |
| Artesunate | DosRST inhibitor | Crystal Violet staining, microscopy | Inhibition assay: concentration dependent reduction in OD value; decrease in biofilm thickness and biofilm density  Destruction assay: no reduction in OD value compared to unexposed control | Belardinelli et al., 2022 |
| OZ277 | DosRST inhibitor | Crystal Violet staining, microscopy | Inhibition assay: concentration dependent reduction in OD value; decrease in biofilm thickness and biofilm density  Destruction assay: no reduction in OD value compared to unexposed control | Belardinelli et al., 2022 |
| OZ439 | DosRST inhibitor | Crystal Violet staining, microscopy | Inhibition assay: concentration dependent reduction in OD value; decrease in biofilm thickness and biofilm density  Destruction assay: no reduction in OD value compared to unexposed control | Belardinelli et al., 2022 |
| HuTipMab | Targets DNA binding proteins (DNABII family) | Microscopy | Reduction in biomass compared to unexposed control | Kurbatfinski et al., 2023 |
| MMV688844 | DNA gyrase inhibitor | CFU determination | Time dependent reduction in CFU value | Negatu et al., 2021 |
| NF1001 | Unknown | CFU determination | MIC: 0.5 µg/mL (planktonic) and 2 µg/mL (biofilm); reduction in log_10_ CFU values of < 0.1 at 16 and 64x MIC | Kaur et al., 2023 |
| Panax quinquefolius | Not described | CFU determination, Crystal Violet staining, microscopy | Concentration dependent reduction in OD value (75-80%) compared to unexposed control; maximum of 7.2 log_10_ reduction in CFU values compared to unexposed control; reduction in biofilm thickness; reduction in biofilm aggregation | He et al., 2023 |
| Curcumin | Hypothesized to target the mycobacterial membrane | Safranin staining , microscopy (live/dead staining) | 100% reduction in OD values compared to unexposed control; increase in dead bacteria staining signal; disruption of biofilm structure | Marini et al., 2018 |
| *Coptic chinensis* | Not described | Safranin staining | Concentration dependent reduction in OD value compared to unexposed control, with a maximal reduction of 0.68 | Tseng et al., 2020 |
| Berberine | Unknown | Safranin staining | Concentration dependent reduction in OD value compared to unexposed control, with a maximal reduction of 0.76 | Tseng et al., 2020 |
| Carvacrol | Not described | Safranin staining, XTT assay | Reduction in OD value and metabolic activity compared to unexposed control | Marini et al., 2019 |
| *Cymbopogon* Flexuos species derived essential oils | Acts on bacterial membrane | Crystal Violet staining | Reduction in OD value compared to unexposed control | Guidolin Rossi et al., 2017 |
